# Treatment of Acidified Blood Using Reduced Osmolarity Mixed-Base Solutions

**DOI:** 10.3389/fphys.2016.00625

**Published:** 2016-12-26

**Authors:** Thomas G. Mason, Jeffrey A. Kraut

**Affiliations:** ^1^Department of Chemistry and Biochemistry, University of California, Los AngelesLos Angeles, CA, USA; ^2^Department of Physics and Astronomy, University of California, Los AngelesLos Angeles, CA, USA; ^3^Medical and Research Services, Veterans Health Administration Greater Los Angeles Area Healthcare SystemLos Angeles, CA, USA; ^4^Division of Nephrology, Veterans Health Administration Greater Los Angeles Area Healthcare SystemLos Angeles, CA, USA; ^5^Membrane Biology Laboratory, David Geffen School of Medicine, University of California, Los AngelesLos Angeles, CA, USA

**Keywords:** acidosis, acidemia, acidotic, strong base, weak base, osmotic stress, spiculation, lysis

## Abstract

We hypothesize that reduced osmolarity mixed-base (ROMB) solutions can potentially serve as customizable treatments for acidoses, going beyond standard solutions in clinical use, such as 1.0 M sodium bicarbonate. Through *in silico* quantitative modeling, by treating acidified canine blood using ROMB solutions, and by performing blood-gas and optical microscopy measurements *in vitro*, we demonstrate that ROMB solutions having a high proportion of a strong base, such as disodium carbonate or sodium hydroxide, can be effective in reducing carbon dioxide pressure PCO_2_ while raising pH and bicarbonate ion concentration without causing significant osmotic damage to red blood cells, which can occur during rapid administration of hypertonic solutions of weak bases. These results suggest that a ROMB solution, which is composed mostly of a strong base, could be administered in a safe and effective manner, when compared to a hypertonic solution of sodium bicarbonate. Because of the reduced osmolarity and the customizable content of strong base in ROMB solutions, this approach differs from prior approaches involving hypertonic solutions that only considered a single molar ratio of strong to weak base. Our calculations and measurements suggest that custom-tailored ROMB solutions merit consideration as potentially efficacious treatments for specific types of acidosis, particularly acute metabolic acidosis and acute respiratory acidosis.

## Introduction

Acute acidosis, lasting from minutes to a few days, can be respiratory or metabolic in nature (DuBose and Hamm, 2002; Kraut and Madias, 2014). Acute respiratory acidosis occurs when the arterial carbon dioxide pressure PCO_2_ rises above its normal range (≈38 mm Hg to ≈42 mm Hg) and the arterial pH falls below its normal range (from ≈7.38 to ≈7.42), usually a result of impaired pulmonary function (Madias et al., 1979; Kraut and Kurtz, 2001; Kraut and Madias, 2010, 2012). Acute metabolic acidosis occurs when serum bicarbonate concentration [${\text{HCO}}_{3}^{-}$] falls below its normal range (24 ± 1.2 mEq/L), usually accompanied by a blood pH below its normal range (Kraut and Madias, 2010). Acute metabolic acidoses are further classified according to the serum anion gap: those in which the anion gap is unchanged (normal anion gap or hyperchloremic acidoses) and those in which the serum anion gap is increased (high anion gap) (Kraut and Kurtz, 2001; Kraut and Madias, 2010). This categorization is not only important for diagnostic purposes but also for decisions about treatment.

Acute acidosis is associated with impaired cellular function and an increase in morbidity and mortality (Gunnerson et al., 2006). This cellular dysfunction is related to a decrease in pH primarily of the interstitial and cellular compartments rather than that of systemic blood (Kraut and Madias, 2012). Presently, eliminating the underlying cause remains the most effective therapy. However, if the underlying cause cannot be eliminated, some clinicians have recommended intravenous administration of basic aqueous solutions (i.e., “administering base”), primarily in the form of sodium bicarbonate (NaHCO_3_), to improve the acid-base milieu of tissues (Kraut and Kurtz, 2001, 2006; Kraut and Madias, 2012, 2016; Velissaris et al., 2015). Nevertheless, sodium bicarbonate administration has failed to improve cellular function or to reduce mortality, even when it improves systemic acid-base balance (Cooper et al., 1990; Forsythe and Schmidt, 2000). This has been ascribed, in part, to exacerbation of intracellular acidosis in response to bicarbonate administration; administered bicarbonate ions ${\text{HCO}}_{3}^{-}$ react with protons to produce carbonic acid, H_2_CO_3._ The carbonic acid can then decompose into CO_2_ and H_2_O. Cells are relatively permeable to CO_2_, which rapidly penetrates them, producing an intracellular respiratory acidosis (Levraut et al., 2001). By contrast to dissolved CO_2_, ${\text{HCO}}_{3}^{-}$ must enter via channels or transporters, and its movement into cells is significantly slower (Roos and Boron, 1981; Levraut et al., 1996; Hulikova and Swietach, 2014). This process, although not inevitable in all circumstances in which bicarbonate is given, appears to be most frequent if the base is given when perfusion of tissues is low (Nielsen et al., 2002).

Other potential adverse effects of sodium bicarbonate administration, including increases in serum osmolality when administered as a hyperosmotic solution and sodium overload, could further undercut its value as a clinical buffer for all causes of acidosis. In current clinical practice, sodium bicarbonate can be administered as a concentrated solution at 8.4%wt, equivalent to a bicarbonate anion concentration [${\text{HCO}}_{3}^{-}$] = 1.0 M. Although bicarbonate can also be administered after dilution or very slowly, such administration would also reduce its capacity to raise blood and tissue pH. As typically supplied, sodium bicarbonate solution is very hypertonic (i.e., up to 2.0 M osmolyte concentration), far beyond the isotonic concentration of osmolyte species in human blood of about 300 mM. If administration of a very hypertonic basic solution occurs too rapidly (e.g., via intravenous rapid bolus injection), cells and other components in the blood could be osmotically damaged before mixing is complete. Avoiding such damage would be essential, particularly in seriously ill patients. Moreover, beyond the potential for osmotic damage, in some studies, administration of a hypertonic solution sodium bicarbonate has been demonstrated to depress cardiovascular function (Huseby and Gumprecht, 1981).

Since the exacerbation of intracellular pH (pH_i_) is presumed to be consequence of generation of excess CO_2_, which during the buffering process enters the cell more rapidly than ${\text{HCO}}_{3}^{-}$, investigators have attempted to develop buffers that would not generate or would even consume excess CO_2_. In the 1950's, solutions of the weak base THAM (i.e., tris-hydroxymethyl aminomethane or “Tris,” p*K*a = 8.07) was introduced as an alternative (Nahas et al., 1998). THAM is a non-carbonate base and consumes protons by virtue of its amino group. Moreover, it penetrates cells and therefore theoretically could raise pH_i_. THAM improves cardiac contractility in individuals with respiratory acidosis (Weber et al., 2000). However, although THAM can raise pH, it has to be eliminated from the body through the kidneys and therefore can create undesirable side effects in individuals who have compromised renal function, a common abnormality in patients who have acute metabolic acidosis. Consequently, although THAM is approved for use in treating acidotic patients, it is administered much less frequently than sodium bicarbonate. Also, rats with lactic acidosis given sodium bicarbonate intravenously were hyperventilated to prevent CO_2_ accumulation; the concomitant hyperventilation led to an increase in cardiac contractility that paralleled the improvement in acid-base balance (Halperin and Kamel, 2010; Kimmoun et al., 2014). The results of studies using THAM or hyperventilation to minimize CO_2_ accumulation highlight the benefits of minimizing CO_2_ generation during the buffering process (Kallet et al., 2000).

In the 1980's, an alternative approach was developed, involving use of a mixed-base solution having fixed 1:1 molar carbonate ratio of 333 mM disodium carbonate (Na_2_CO_3_), a strong base, and 333 mM sodium bicarbonate, yielding a 1.67 M total osmolyte concentration (Filley and Kindig, 1985; Bersin and Arieff, 1988). Termed “Carbicarb,” this solution was introduced because of its potential to raise pH while at the same time inhibiting significant generation of CO_2_. Carbicarb (measured pH = 9.6) was significantly more basic than sodium bicarbonate, yet it was still very hypertonic. While not explained so directly in the original publications, compared to our simple explanation below, introducing disodium carbonate into the base's composition can reduce dissolved CO_2_ levels. A divalent carbonate anion ${\text{CO}}_{3}^{2-}$ can readily react with a hydronium ion (H_3_O^+^) in a partial neutralization reaction, yielding ${\text{HCO}}_{3}^{-}$ and a water molecule. Alternatively, ${\text{CO}}_{3}^{2-}$ can react with a water molecule (i.e., via hydrolysis), yielding a bicarbonate ion and a hydroxide ion (OH^−^). This generated hydroxide ion, in turn, can react with a dissolved CO_2_ molecule, thereby consuming it and producing a bicarbonate ion. Thus, the addition of ${\text{CO}}_{3}^{2-}$ to a neutral or acidic solution containing dissolved CO_2_ leads to the net production of bicarbonate ions and the net consumption of CO_2_. Moreover, our explanation of the fundamental mechanism for this effect is general and suggests that any strong base, which is capable of generating hydroxide ions at sufficiently high concentrations, could potentially be used to reduce dissolved CO_2_ levels. Obviously, anions of strong bases also have the potential to participate in other reactions, including deprotonating proteins, lipids, and other molecules that they encounter, thereby causing undesirable caustic damage to tissues, so ideally such highly reactive anions would be confined to react only with small and very simple molecules and ions, but not larger structures in the blood, vasculature, and other tissues.

Subsequent *in vitro* experiments, in which Carbicarb or sodium bicarbonate was added to acidified blood in a closed system (so as to identify production of CO_2_), revealed that addition of sodium bicarbonate increased PCO_2_, whereas the addition of Carbicarb led to little or no increase in PCO_2_ (Shapiro et al., 1995). *In vivo* studies in animals which have acute lactic acidosis revealed that Carbicarb improved systemic acid-base parameters, raised intracellular pH and improved cardiac function, whereas sodium bicarbonate did not improve cardiac function or improve intracellular pH (Bersin and Arieff, 1988). By contrast, Carbicarb, when administered to humans who had mild metabolic acidosis (serum [${\text{HCO}}_{3}^{-}$] ≈ 20 mEq/L) failed to provide more benefit than sodium bicarbonate (Leung et al., 1994). Although, this human study had a very limited scope, as a consequence of this result, Carbicarb was never adopted in clinical practice. However, because Carbicarb was reported to be effective in the treatment of acute acidosis in limited animal studies, we postulate that other mixed base solutions at reduced osmolarity compared to Carbicarb, might also be effective and deserve consideration.

The cellular dysfunction of acute acidosis, whether it be metabolic or respiratory in origin, is related to reductions in the intracellular and interstitial pH of vital tissues, primarily that of the myocardium (Huang et al., 1995; Vaughan-Jones et al., 2009; Schroeder et al., 2010). The failure of sodium bicarbonate to improve cellular dysfunction is directly related to its inability to consistently improve the acid-base milieu of the compartments (Kraut and Madias, 2012). Indeed, in several experimental studies it has been shown to exacerbate the acidosis (Kindig and Filley, 1983; Levraut et al., 1996; Forsythe and Schmidt, 2000). So, in light of these studies, it therefore seems reasonable to postulate that development of alternative base solutions, tailored for the treatment of specific types and severities of acute acidoses, could potentially reduce acidification of vital tissues and improve cellular function.

In the present paper, we present theoretical calculations of the physical properties of mixed base solutions containing different proportions of weak and strong bases. These results lead us to introduce the concept of reduced osmolarity mixed base (ROMB) solutions. ROMB solutions can contain a greater proportion of a strong base than present in Carbicarb but at a lower osmolarity, which reduces its potential for causing both caustic and osmotic damage. At the same time, the higher proportion of the strong base (e.g., 3:1 strong:weak) offers the advantage of consuming more CO_2_, on a per molar basis of administered solution, than simple 1:1 Carbicarb.

As a preliminary exploration of this hypothesis, we have tested the ability of ROMB solutions to improve the acid-base balance of acidified canine blood by adding one of a variety of base solutions to determine their impact on plasma bicarbonate, blood pH, and PCO_2_. Overall, a ROMB solution having a 3:1 ratio of Na_2_CO_3_: NaHCO_3_ performs well in raising plasma pH and [${\text{HCO}}_{3}^{-}$] while reducing PCO_2_. Interestingly, ROMB solutions of sodium hydroxide NaOH with NaHCO_3_ also offer a similar ability to raise plasma pH and [${\text{HCO}}_{3}^{-}$] while reducing PCO_2_ and lowering the sodium load. Thus, ROMB solutions appear to be worthy of further attention for *in vivo* testing as possible alternative bases for the treatment of acute acidoses. Future studies will be necessary to determine if such compositions suggested by these *in silico* and *in vitro* results will lead to improved outcomes in animals and humans.

## Materials and methods

### Modeling and computation of chemical properties of mixed-base solutions

#### Aqueous solutions of sodium bicarbonate and disodium carbonate

We solve for the equilibrium concentrations of ionic species in water after the addition of sodium bicarbonate and disodium carbonate using the full, exact set of equilibrium equations that include charge neutrality. This approach leads to a set of four equations and four unknowns, which can be solved by finding the appropriate zero of a quartic polynomial; we numerically solve this quartic equation using Mathematica (Wolfram Research Inc.).

When sodium bicarbonate NaHCO_3_ is dissolved in neutral water, it dissociates:

$$\begin{array}{l} {\text{NaHCO}}_{3}\left(s\right)\to {\text{Na}}^{+}\left(aq\right)+{\text{HCO}}_{3}^{-}\left(aq\right). \end{array}$$

The initial concentration of the bicarbonate anion ${\text{HCO}}_{3}^{-}$ is given by *y*_0_ = [${\text{HCO}}_{3}^{-}$]_0_. When disodium carbonate Na_2_CO_3_ is dissolved in neutral water, it also dissociates:

$$\begin{array}{l} {\text{Na}}_{2}{\text{CO}}_{3}\left(s\right)\to {2\text{Na}}^{+}\left(aq\right)+{\text{CO}}_{3}^{2-}\left(aq\right). \end{array}$$

The initial concentration of the carbonate anion ${\text{CO}}_{3}^{2-}$ is given by *z*_0_ = [${\text{CO}}_{3}^{2-}$]_0_. Conservation of matter requires that the initial concentration of sodium [Na^+^]_0_ is:

$$\begin{array}{l} {\left[{\text{Na}}^{+}\right]}_{0}={\left[{\text{HCO}}_{3}^{-}\right]}_{0}+2{\left[{\text{CO}}_{3}^{2-}\right]}_{0.} \end{array}$$

Since Na^+^ is ineffective in acid-base chemistry, the equilibrium concentration of Na^+^ is:

$$\begin{array}{l} \left[{\text{Na}}^{+}\right]={\left[{\text{Na}}^{+}\right]}_{0}={\left[{\text{HCO}}_{3}^{-}\right]}_{0}+2{\left[{\text{CO}}_{3}^{2-}\right]}_{0.} \end{array}$$

Given [${\text{HCO}}_{3}^{-}$]_0_ and [${\text{CO}}_{3}^{2-}$]_0_ through the amounts of sodium bicarbonate and disodium carbonate added to neutral water, which has *h*_0_ = [H_3_O^+^]_0_ = 10^−7^ M, we set up a set of simultaneous equations and solve for the equilibrium pH and concentrations of the various ionic species.

The hydronium ion concentration at equilibrium is defined to be *h* = [H_3_O^+^], where *K*_w_ = 10^−14^ is the equilibrium constant associated with water auto-ionization, and [H_3_O^+^][OH^−^] = *K*_w_, so [OH^−^] = *K*_w_/*h*. To simplify notation, we assign the following variables to represent the equilibrium concentrations of the carbonate species: *x* = [H_2_CO_3_], *y* = [${\text{HCO}}_{3}^{-}$], and *z* = [${\text{CO}}_{3}^{2-}$]. We assume that no carbonic acid is present initially, so we set *x*_0_ = [H_2_CO_3_]_0_ = 0. We write equations for conservation of matter of the carbonate species, for charge neutrality, and for the deprotonation reactions of carbonic acid and the bicarbonate anion, respectively. Conservation of carbonate species at equilibrium means [${\text{HCO}}_{3}^{-}$]_0_ + [${\text{CO}}_{3}^{2-}$]_0_ = [${\text{HCO}}_{3}^{-}$] + [H_2_CO_3_] + [${\text{CO}}_{3}^{2-}$], so,

(1) $$\begin{array}{r} x=\left(y_{0}-y\right)+\left(z_{0}-z\right). \end{array}$$

Charge neutrality at equilibrium yields: [Na^+^] + [H_3_O^+^] = [${\text{HCO}}_{3}^{-}$] + 2[${\text{CO}}_{3}^{2-}$] + [OH^−^], so:

(2) $$\begin{array}{r} \left(K_{\text{w}}/h\right)-h=\left(y_{0}-y\right)+2\left(z_{0}-z\right). \end{array}$$

Deprotonation of carbonic acid proceeds according to the following equilibrium reaction:

$$\begin{array}{l} {{\text{H}}_{2}\text{CO}}_{3}\left(aq\right)+{\text{H}}_{2}\text{O}\left(l\right)↔{{\text{H}}_{3}\text{O}}^{+}\left(aq\right)+{\text{HCO}}_{3}^{-}\left(aq\right). \end{array}$$

The law of mass action requires *K*a_1_ = [H_3_O^+^] [${\text{HCO}}_{3}^{-}$]/[H_2_CO_3_] = 4.3 × 10^−7^, so:

(3) $$\begin{array}{r} K{\text{a}}_{1}=hy/x. \end{array}$$

Deprotonation of the bicarbonate anion proceeds according to:

$$\begin{array}{l} {\text{HCO}}_{3}^{-}\left(aq\right)+{\text{H}}_{2}\text{O}\left(l\right)↔{\text{H}}_{3}{\text{O}}^{+}\left(aq\right)+{\text{CO}}_{3}^{2-}\left(aq\right) \end{array}$$

The law of mass action requires *K*a_2_ = [H_3_O^+^] [${\text{CO}}_{3}^{2-}$]/[${\text{HCO}}_{3}^{-}$] = 4.8 × 10^−11^, so:

(4) $$\begin{array}{r} K{\text{a}}_{2}=hz/y. \end{array}$$

In this calculation, we use *K*a_1_ = 4.3 × 10^−7^ for carbonic acid deprotonation, which effectively incorporates the equilibrium between dissolved CO_2_ and H_2_CO_3_, and *K*a_2_ = 4.8 × 10^−11^ for deprotonation of the bicarbonate anion at room temperature.

Thus, if all initial conditions are specified, Equations (1–4), contain four unknowns *h*, *x*, *y*, and *z*, and these equations can be solved exactly to provide equilibrium concentrations. We can rewrite Equation 3 as *x* = *hy/K*a_*1*_ and Equation 4 as *z* = *K*a_*2*_*y/h*. Substituting these relationships into Equation (1) yields:

$$\begin{array}{l} hy/K{\text{a}}_{1}=\left(y_{0}-y\right)+\left[z_{0}-\left(K{\text{a}}_{2}\;y/h\right)\right], \end{array}$$

which can be solved to obtain *y* in terms of *h*:

(5) $$\begin{array}{r} y=\left(y_{0}+z_{0}\right)/\left[1+\left(h/K{\text{a}}_{1}\right)+\left(K{\text{a}}_{2}/h\right)\right]. \end{array}$$

Substituting relationships for *z* into Equation (2) yields:

$$\begin{array}{l} \left(K_{\text{w}}/h\right)-h=\left(y_{0}-y\right)+2\left(z_{0}-K{\text{a}}_{2}\;y/h\right) \end{array}$$

which can also be solved to obtain *y* in terms of *h*:

(6) $$\begin{array}{r} y=\left[y_{0}+2z_{0}+h-\left(K_{\text{w}}/h\right)\right]/\left[1+\left(2K{\text{a}}_{2}/h\right)\right]. \end{array}$$

The right hand sides of Equations (5) and (6) for *y* must be equal to ensure self-consistency, so:

$$\begin{array}{l} {}[1+(2K{\text{a}}_{2}/h)](y_{0}+z_{0})=[1+(h/K{\text{a}}_{1})+(K{\text{a}}_{2}/h)][y_{0}+2z_{0}\\ \;+\;h-(K_{\text{w}}/h)]. \end{array}$$

Expanding and collecting terms having different powers of *h* yields a 4th order (i.e., quartic) polynomial in *h*, which simplifies to:

(7) $$\begin{array}{l} h^{4}/K{\text{a}}_{1}+[1+(y_{0}+2z_{0})/K{\text{a}}_{1}]h^{3}+[K{\text{a}}_{2}-(K_{\text{w}}/K{\text{a}}_{1})+z_{0}]h^{2}\\ \;-\;(K_{\text{w}}+K{\text{a}}_{2}\;y_{0})h-(K{\text{a}}_{2}\;K_{\text{w}})=0. \end{array}$$

Although somewhat complex, quartic equations can be solved. From among the four roots, which can include imaginary numbers, we select the physical solution of Equation 7 for a unique value of *h* = [H_3_O^+^] that is positive and real. Other imaginary or negative solutions are discarded. Once *h* is known, *y*, *x*, and *z* can also be calculated. Using nested loops in the Mathematica program, we calculate a set of equilibrium results for a variety of initial concentrations of NaHCO_3_ and Na_2_CO_3_. In the calculations, we designate the total carbonate concentration to be *C*_tot_ = [${\text{HCO}}_{3}^{-}$]_0_ + [${\text{CO}}_{3}^{2-}$]_0_ and we designate the initial ratio of bicarbonate to carbonate ions to be *R* = [${\text{HCO}}_{3}^{-}$]_0_/[${\text{CO}}_{3}^{2-}$]_0_. Our calculations assume ideal activities for the ionic species even at larger concentrations, so they should be taken as a guide for revealing trends, and we would not necessarily expect these calculated values to match experimental values exactly as the solutions become more concentrated and non-ideal activities play more important roles.

The value of *x* calculated in the previous equations actually accounts for the sum of the dissolved carbonic acid plus dissolved carbon dioxide, so we define [H_2_CO_3_]_calc_ = *x* (combined). To separate out the contributions to the different neutral species, CO_2_(*aq*) and H_2_CO_3_(*aq*), it is necessary to consider the so-called “hydration” equilibrium reaction of CO_2_ in water:

$$\begin{array}{l} {\text{CO}}_{2}\left(aq\right)+{\text{H}}_{2}\text{O}\left(l\right)↔{\text{H}}_{2}{\text{CO}}_{3}\left(aq\right). \end{array}$$

The law of mass action requires *K*_hyd_ = [H_2_CO_3_]_act_/[CO_2_]_act_, where *K*_hyd_ = 1.7 × 10^−3^ at room temperature, [CO_2_]_act_ is the actual concentration of CO_2_ in the solution, and [H_2_CO_3_]_act_ is the actual concentration of H_2_CO_3_ in the solution. Since

(8) $$\begin{array}{r} x={\left[{{\text{H}}_{2}\text{CO}}_{3}\right]}_{\text{calc}}={\left[{{\text{H}}_{2}\text{CO}}_{3}\right]}_{\text{act}}+{\left[{\text{CO}}_{2}\right]}_{\text{act}}, \end{array}$$

we solve for [CO_2_]_act_ = [H_2_CO_3_]_calc_/(1 + *K*_hyd_) and [H_2_CO_3_]_act_ = [H_2_CO_3_]_calc_ − [CO_2_]_act_.

Once [CO_2_]_act_ has been determined then Henry's Law can be used to calculate the partial pressure of CO_2_, commonly known as PCO_2_:

(9) $$\begin{array}{r} {\text{PCO}}_{2}=K_{\text{H}}{\left[{\text{CO}}_{2}\right]}_{\text{act},} \end{array}$$

where *K*_H_ = 2.23 × 10^4^ mm Hg/M is Henry's Law value for CO_2_ in H_2_O at room temperature. Pressures are then conveniently expressed in the typical format of mm Hg when [CO_2_]_act_ is specified in molar units.

#### Aqueous solutions of sodium bicarbonate and sodium hydroxide

A certain amount of sodium bicarbonate is added to neutral water, yielding an initial concentration of the bicarbonate anion [${\text{HCO}}_{3}^{-}$]_0_ = *y*_0_. A certain amount of sodium hydroxide is added to the same solution, yielding an initial concentration of the hydroxide anion [OH^−^]_0_ = *b*_0_, where we assume that *b*_0_ = [OH^−^]_0_ ≫ 10^−7^ M (i.e., the concentration of hydroxide anions in neutral water). The initial concentrations of the carbonate anion and carbonic acid are also zero: *x*_0_ = [H_2_CO_3_]_0_ = 0 and *z*_0_ = [${\text{CO}}_{3}^{2-}$]_0_ = 0. The sodium bicarbonate dissociates in water as described previously, and the sodium hydroxide dissociates according to:

$$\begin{array}{l} \text{NaOH}\left(s\right)\to {\text{Na}}^{+}\left(aq\right)+{\text{OH}}^{-}\left(aq\right). \end{array}$$

The initial concentration of the sodium cation is: [Na^+^]_0_ = [${\text{HCO}}_{3}^{-}$]_0_ + [OH^−^]_0_ = *y*_0_ + *b*_0_. Since sodium is ineffective in acid-base chemistry, the equilibrium concentration of the sodium cation is simply the initial concentration: [Na^+^] = [Na^+^]_0_ = *y*_0_ + *b*_0._ Conservation of carbonate species requires: [${\text{HCO}}_{3}^{-}$]_0_ = [H_2_CO_3_] + [${\text{HCO}}_{3}^{-}$] + [${\text{CO}}_{3}^{2-}$], so:

(10) $$\begin{array}{r} y_{0}=x+y+z. \end{array}$$

Charge neutrality requires: [Na^+^] + [H_3_O^+^] = [${\text{HCO}}_{3}^{-}$] + 2[${\text{CO}}_{3}^{2-}$] + [OH^−^], implying:

(11) $$\begin{array}{r} \left(K_{\text{w}}/h\right)-h=b_{0}+y_{0}-y-2z. \end{array}$$

For the two reactions describing the carbonate equilibria, the respective laws of mass action are given by Equations (3) and (4). Substituting these equations into Equation (10) for the conservation of carbonate species yields:

(12) $$\begin{array}{r} y=y_{0}/\left[1+\left(h/K{\text{a}}_{1}\right)+\left(K{\text{a}}_{2}/h\right)\right], \end{array}$$

and into Equation 11 for the charge neutrality equation yields:

(13) $$\begin{array}{r} y=\left[y_{0}+b_{0}+h-\left(K_{\text{w}}/h\right)\right]/\left(1+2K{\text{a}}_{2}/h\right). \end{array}$$

Self-consistency requires that both expressions for *y* in terms of *h* must be equal, so:

$$\begin{array}{l} \left[y_{0}+b_{0}+h-(K_{\text{w}}/h)][1+(h/K{\text{a}}_{1})+(K{\text{a}}_{2}/h)\right]\\ \;=y_{0}(1+2K{\text{a}}_{2}/h). \end{array}$$

From this, we obtain a different quartic equation in the hydronium ion concentration valid for mixtures of the weak base, NaHCO_3_, with the strong base, NaOH:

(14) $$\begin{array}{l} \left[h^{2}+h(y_{0}+b_{0})-K_{\text{w}}][(h^{2}/K{\text{a}}_{1})+h+K{\text{a}}_{2}\right]\\ \;-\;y_{0}(h^{2}+2K{\text{a}}_{2}h)=0. \end{array}$$

We solve this quartic equation using Mathematica for various initial concentrations of sodium bicarbonate, *y*_0_, and sodium hydroxide, *b*_0_. Using the positive real values of *h* for the solution, which provides the equilibrium [H_3_O^+^], we obtain the equilibrium concentrations *y* = [${\text{HCO}}_{3}^{-}$] using Equation (12), *z* = [${\text{CO}}_{3}^{2-}$] using Equation (4), and *x* = [H_2_CO_3_] using Equation (3). Using the CO_2_ hydration equilibrium reaction and Henry's law in Equations (8) and (9), respectively, we then calculate the equilibrium carbon dioxide pressure PCO_2_ from [CO_2_]_act_.

### Experimental materials and methods

#### Acidification of canine blood

Canine blood was obtained from Animal Blood Resources International (Dixon, CA) and stored at 4°C. Prior to shipment, this blood was treated by the supplier with a citric anticoagulant (CA) (Hess, 2006), citric phosphate dextrose adenine CPDA-1, at a ratio of about 1 part CPDA-1 solution to 9 parts blood. Consequently, this CA-treated blood was pre-acidified when received. Because blood ages during storage, we label blood samples used on different dates, well within the supplier's use-by date, and measure their baseline blood-gas parameters, which can change over time. We label a first CA-treated blood sample having a measured pH = 6.98 as CA-K9-1 and a second older CA-treated blood sample having a measured pH = 6.90, slightly more acidic, as CA-K9-2. Subsequently, a third CA-treated blood sample, CA-K9-3, was obtained fresh from the supplier; its measured pH = 7.00 is very close to that of the first fresh blood sample. For some experiments, to keep BGA parameters of base-treated acidified blood within the measurement range of our BGA instrument, we have further treated the canine blood to make it more acidic using an aqueous solution of hydrochloric acid (HCl). Sample HCl-CA-K9-2, having a measured pH = 6.62, was made by mixing 1 part of a 150 mM solution of HCl with 9 parts of CA-K9-2, and sample HCl-CA-K9-3, having a similar measured pH = 6.66, was obtained in the same way using CA-K9-3.

#### Measuring pH and blood-gas parameters of blood and blood-base mixtures

To measure how new types of ROMB solutions affect blood chemistry and to identify their compositions and concentrations that could be suitable for treating acute acidosis, we have designed and carried out experiments using canine blood that has been pre-acidified using a citric anticoagulant. The primary blood chemistry tests have been carried out using an IDEXX VetStat Blood Gas Analyzer (BGA) and single-use IDEXX Electrolyte 8+ cassettes. The VetStat BGA has been calibrated using all standard reference cassettes, passing the necessary reference tests, and it was also calibrated using Levels 1, 2, and 3 OptiCheck fluids, passing all calibration fluid tests successfully, thereby ensuring accuracy of our subsequent measurements. The BGA directly measures pH and PCO_2_ and uses these values to determine the concentration of the bicarbonate ion [${\text{HCO}}_{3}^{-}$]. The pH range of the BGA has a lower limit of ≈6.4 and an upper limit of ≈7.8, and the PCO_2_ has a lower limit of ≈10 mm Hg. The BGA also directly measures [Na^+^], [K^+^], and [Cl^−^]. All BGA measurements have been made at a temperature *T* = 37°C. When mixing, we used glass containers, syringes, and capillaries as well as stainless steel needles. The residence time was short enough and transfer to the VetStat BGA device rapid enough that CO_2_ diffusion out of the mixture was not significant (i.e., our measurements are effectively performed in the closed system limit). After calibration, replicate testing of the BGA instrument's precision on separate samples of CA-treated blood gave the following measured absolute and percent standard deviations: pH = ±0.05 (± 0.8%), PCO_2_ = ±7.6 mm Hg (±9.7%), [${\text{HCO}}_{3}^{-}$] = ±0.4 mM (±2.5%), [Na^+^] = ±1.1 mM (±0.8%), [K^+^] = ±0.5 mM (±12.9%), and [Cl^−^] = ±1.7 mM (±1.7%). These small uncertainties were obtained after three trials, indicating the high precision of the BGA.

In addition, some pH-titration measurements have been made using an Accumet pH meter equipped with an electrode probe, suitable for small volumes, connected to a computer. For titrations made using the pH meter, the volume dispense rate of the base solution has been controlled using a computer-controlled syringe-pump dispenser and a stir bar is used to mix the added solution with the blood.

Acidified blood samples were treated with different base solutions by adding 1 part of the base solution (e.g., 100 μL) to 9 parts of acidified canine blood (e.g., 900 μL) and mildly agitating for 10–20 s. The base-treated blood was then injected using a syringe into a heparin-treated capillary tube of 200 microliter capacity. The capillary tube was inserted into the aspiration nozzle of the Electrolyte 8+ cassette of the VetStat BGA, and the BGA then tested the blood sample and recorded the results. For each mixture, the entire procedure took less than 5 minutes from mixing to loading of the BGA. These conditions effectively yielded BGA results for a passive closed system in which active processes, such as respiration, were absent. A small amount of the base-treated blood was transferred onto a heparin-treated glass slide with a No. 1 coverslip for viewing using the optical microscope. Into some base solutions that have lower ion concentrations, we added sodium chloride, NaCl, in order to raise their osmolarities without affecting pH.

#### Optical microscopy of acidified blood and treated blood

Optical microscopy was performed with a National Optical brightfield microscope using a 40 × objective and a digital camera (Flea2 by Point Grey Research). The mixtures of solutions and treated blood were made in low-flow conditions; harsh high-flow mixing methods were purposefully avoided so changes in observed structures could be primarily associated with different solution chemistries. The image size (i.e., distance scale) has been calibrated using a 100 line-per-mm reticle. Simple pH measurements using the pH meter and optical microscope were carried out at room temperature *T* ≈ 23°C. Real-time blood titration experiments have been performed with an automated Hamilton syringe delivery system using the Accumet pH meter, controlled by custom-written LabVIEW software.

## Results

### Computer calculations of properties of mixed-base solutions

**Figure 1:**
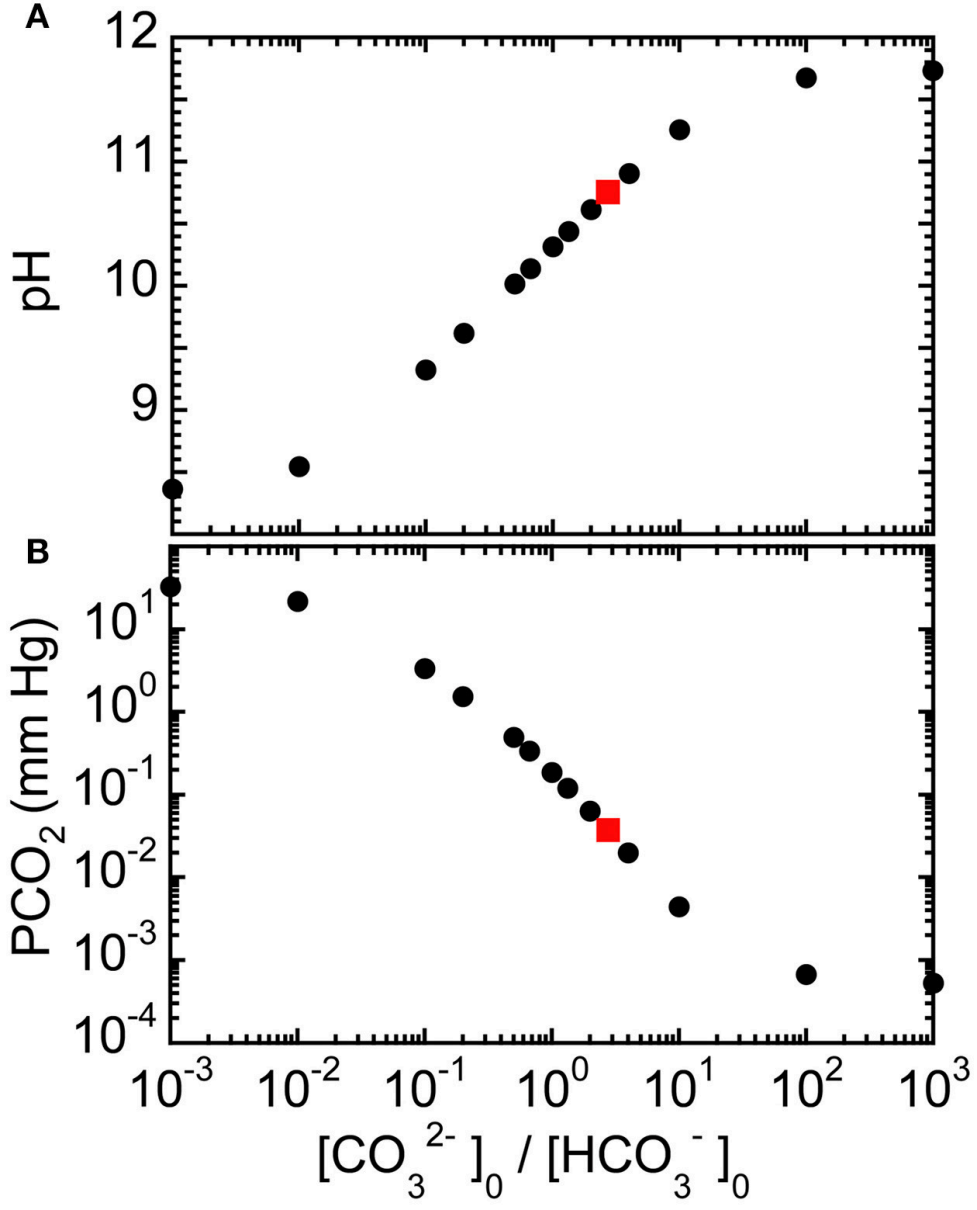
**Predicted equilibrium properties of reduced osmolarity mixed-base (ROMB) solutions composed of NaHCO_3_ and Na_2_CO_3_**. **(A)** pH and **(B)** PCO_2_ as a function of the ratio of the initial concentration of carbonate anions [${\text{CO}}_{3}^{2-}$]_0_ to the initial concentration of bicarbonate anions [${\text{HCO}}_{3}^{-}$]_0_, where the total concentration of carbonate species has been fixed at 150 mM. Gray squares mark the idealized predicted values of pH ≈ 10.8 and PCO_2_ ≈ 0.03 mm Hg for a 3:1 Na_2_CO_3_: NaHCO_3_ ROMB solution.

We solve for the equilibrium pH and ion concentrations in a basic aqueous solution after dissolving known quantities of sodium bicarbonate and of disodium carbonate into neutral water (see Materials and Methods). The predicted equilibrium pH values for a NaHCO_3_: Na_2_CO_3_ base solution at a 0.15 M fixed total carbonate concentration *C*_tot_ = [${\text{HCO}}_{3}^{-}$]_0_ + [${\text{CO}}_{3}^{2-}$]_0_ for different ratios of initial [${\text{CO}}_{3}^{2-}$]_0_/[${\text{HCO}}_{3}^{-}$]_0_ are shown in Figure 1A. Here, we have chosen *C*_tot_ = 0.15 M to be much lower than Carbicarb, which has *C*_tot_ = 0.667 M, so the resulting ROMB solution would reduce the potential for adverse osmotic effects on blood cells. In the limit of small [${\text{CO}}_{3}^{2-}$]_0_/[${\text{HCO}}_{3}^{-}$]_0_, corresponding to a nearly pure sodium bicarbonate solution, the resulting predicted pH ≈ 8.4 agrees well with the known value of this weak base (Oxtoby et al., 2012). As a greater proportion of Na_2_CO_3_ is used in the mixture, the pH increases until it saturates near ≈ 11.7, corresponding to a nearly pure disodium carbonate solution. In Figure 1B, the corresponding results for PCO_2_ at *C*_tot_ = 0.15 M are shown over a wide range of mixing ratios. As a greater proportion of the strong base Na_2_CO_3_ is used in the mixture, PCO_2_ is significantly reduced. So, one may infer that mixed-base solutions made using more Na_2_CO_3_ would tend to reduce PCO_2_ levels if administered. Although, the 0.15 M solution has a reduced osmolarity compared to sodium bicarbonate and Carbicarb, it is possible to achieve about the same pH-raising capacity as a standard treatment solution of 1.0 M sodium bicarbonate for the 3:1 mixture having [${\text{CO}}_{3}^{2-}$]_0_/[${\text{HCO}}_{3}^{-}$]_0_ ≈ 3. This 3:1 mixture can be made by mixing 3 parts of 150 mM Na_2_CO_3_ solution with 1 part of 150 mM NaHCO_3_ solution, yielding [${\text{CO}}_{3}^{2-}$]_0_ = 112.5 mM and [${\text{HCO}}_{3}^{-}$]_0_ = 37.5 mM, and a total osmolarity of about 412.5 mM, including sodium ions.

**Figure 2:**
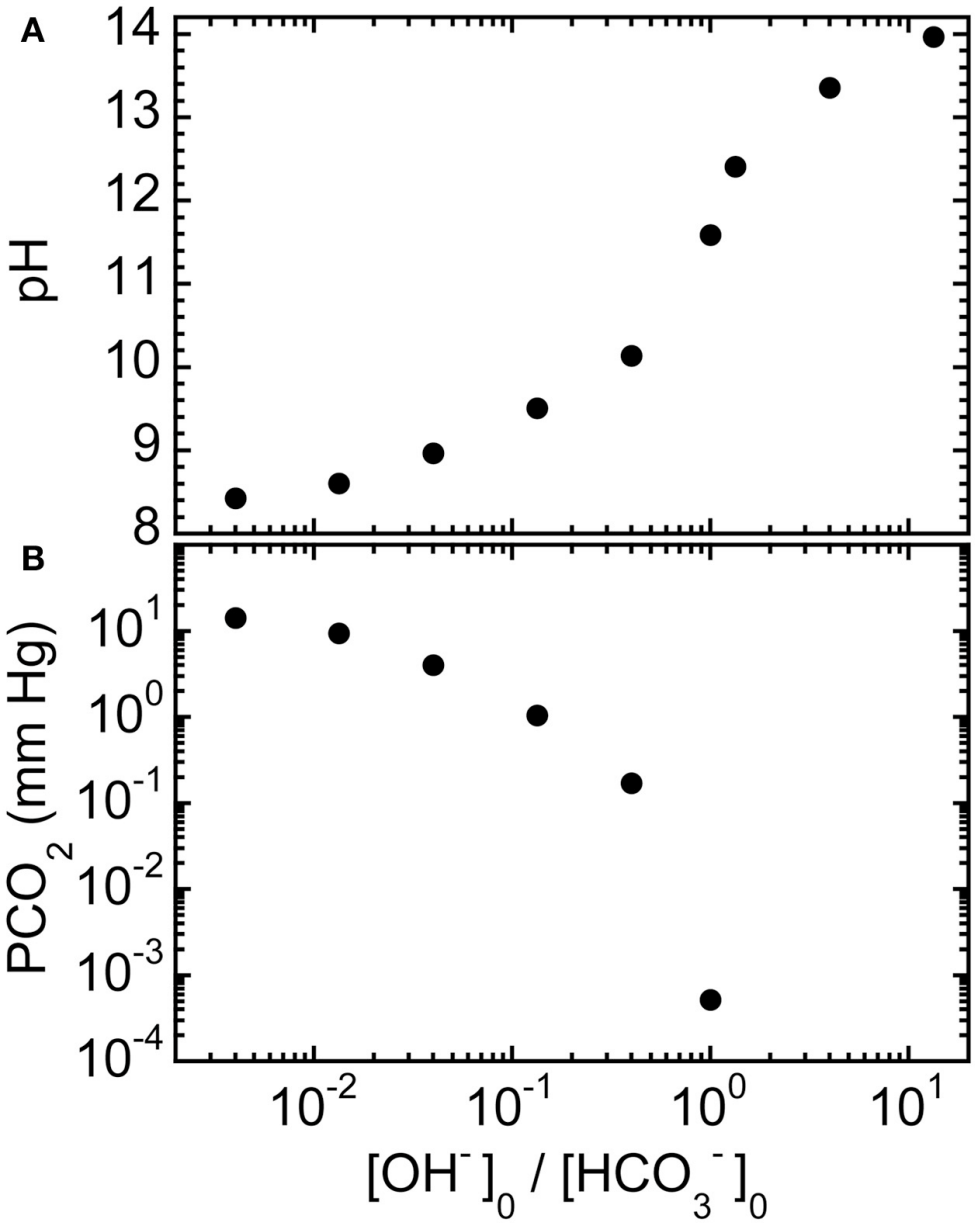
**Predicted equilibrium properties of ROMB solutions composed of NaHCO_3_ and NaOH. (A)** pH and **(B)** PCO_2_ of ROMB solutions resulting from mixing different proportions of a 150 mM NaOH solution to a 150 mM NaHCO_3_ solution, expressed by the ratio of the initial concentration of hydroxide ions [OH^−^]_0_ divided by the initial concentration of bicarbonate anions [${\text{HCO}}_{3}^{-}$]_0_. The total initial concentration of basic species is fixed at 150 mM. For ratios greater than 1, PCO_2_ has a vanishingly small value and is not shown.

We have also calculated equilibrium values of pH and PCO_2_ for 150 mM mixed-base solutions that are made using NaHCO_3_ and NaOH for different ratios of initial concentrations [OH^−^]_0_/[${\text{HCO}}_{3}^{-}$]_0_ (see Materials and Methods), as shown in Figures 2A,B, respectively. Although the trends are similar to what has been shown in Figure 1, the use of sodium hydroxide can further reduce PCO_2_ while providing an even larger increase in pH and also keeping the final equilibrium [Na^+^] lower than for corresponding mixtures of sodium bicarbonate and disodium carbonate. Thus, theory predicts that the using other types of strong bases, not just disodium carbonate, in mixed-base solutions could potentially be useful for custom-tailoring the base solution to treat different types of acute acidoses.

### Blood gas and pH analysis of acidified blood treated by mixed-base solutions

Experimental results of BGA measurements of solutions that have been mixed with acidified canine blood samples yield responses as a function of types, concentrations, and relative proportions of species (see Tables 1–6). For instance, sodium bicarbonate raises pH relatively little compared to strong bases at similar molarity, yet it also elevates PCO_2_ substantially, which is typically undesirable in treatment. By contrast, base-treatment solutions of strong bases, such as Na_2_CO_3_ and NaOH, raise pH by about 0.5 pH units. We find that Na_2_CO_3_ is highly effective in raising pH and [${\text{HCO}}_{3}^{-}$] while lowering PCO_2_ substantially. Similarly, NaOH, which is a strong base yet contains no carbonate species, is also highly effective in raising pH. Moreover, 150 mM NaOH solution lowers PCO_2_ even more than the same molarity solution of Na_2_CO_3_. Thus, solutions containing NaOH could potentially be used instead of those containing Na_2_CO_3_ in certain situations to control or reduce PCO_2_ if desired. We also find that a ROMB solution composed of 75 mM Na_2_CO_3_ + 75 mM NaOH, which has 150 mM total of strong base species, raises pH by about the same amount as either 150 mM pure Na_2_CO_3_ or 150 mM pure NaOH solutions, yet results in values of PCO_2_ and [${\text{HCO}}_{3}^{-}$] that are intermediate between those of the two pure base solutions. Thus, by adjusting the proportion of two strong bases, Na_2_CO_3_ and NaOH, in a ROMB solution, it is possible to control plasma [${\text{HCO}}_{3}^{-}$] in a desirable range while increasing plasma pH and decreasing PCO_2_.

**Table 1:** **Responses of CA acidified canine blood to added solutions**.

**Added solution**	**Conc. (mM)**	**pH**	**PCO_2_ (mm Hg)**	**[${\text{HCO}}_{3}^{-}$] (mM)**	**[Na^+^] (mM)**	**[K^+^] (mM)**	**[Cl^−^] (mM)**
None	0	6.90	87	15.8	148	4.2	98
Na_2_CO_3_	150	7.57	43	37.1	164	3.4	103
NaOH	150	7.47	23	15.3	147	3.6	98
Na_2_CO_3_ + NaOH (1:1)	75 + 75 = 150 tot	7.47	36	24.0	156	3.5	100
HCl	150	6.62	113	10.8	128	3.8	94
*(1 part solution: 9 parts CA-K9-3)*							
None	0	7.00	73	16.6	150	3.3	101
NaOH NaCl	150 75	7.54	21	16.4	155	2.9	105
Tris soln	150	7.50	27	19.5	135	2.9	98
Tris soln NaCl	150 75	7.50	26	19.1	144	2.9	104
Tris buffer	150	7.18	41	14.4	134	3.0	100

**Figure 3:**
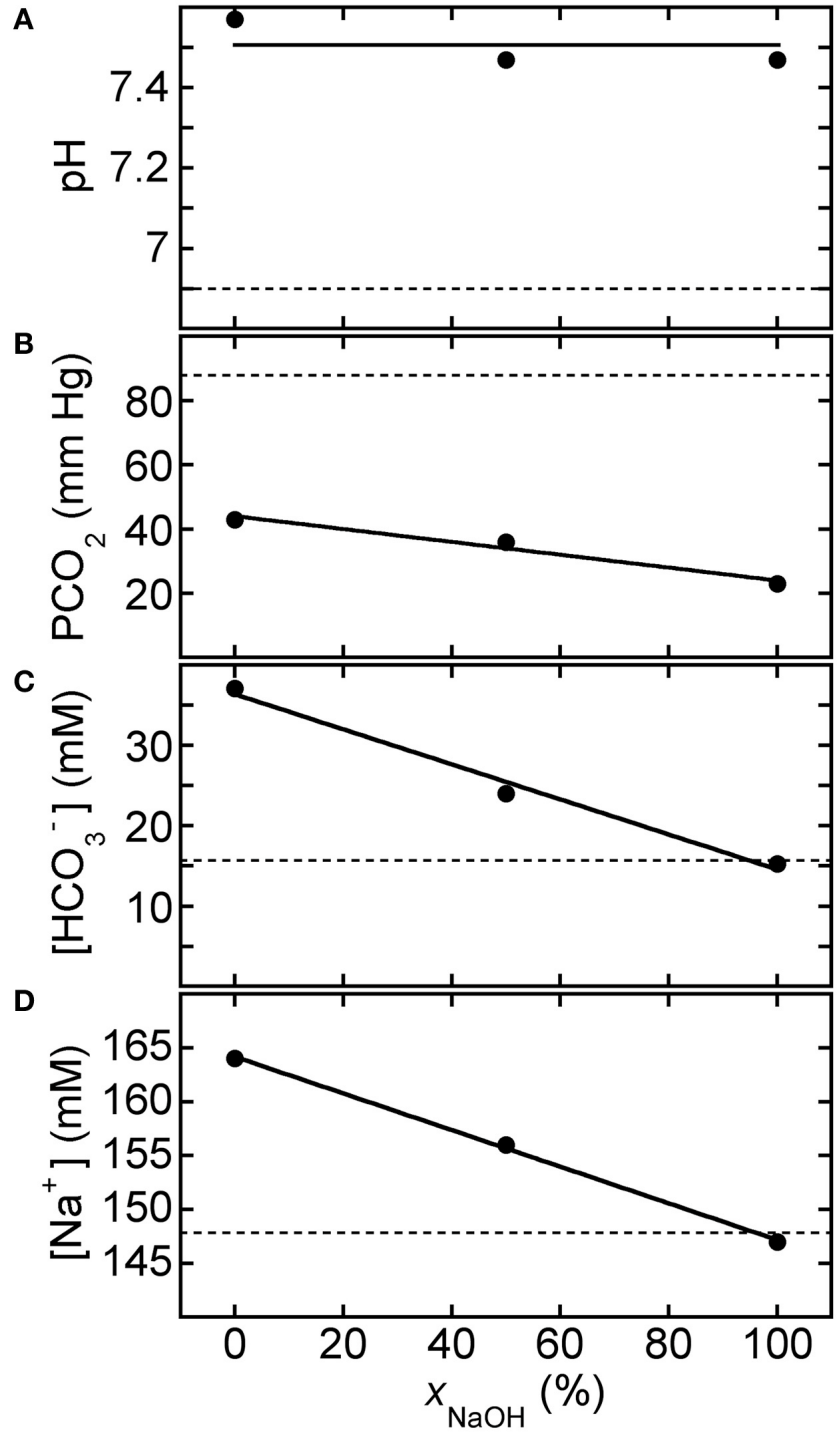
**Response of acidified canine blood to ROMB solutions composed of Na_2_CO_3_ and NaOH**. Measured blood gas parameters **(A)** pH, **(B)** PCO_2_, **(C)** [${\text{HCO}}_{3}^{-}$], and **(D)** [Na^+^] of citric acid (CA) acidified canine blood CA-K9-2 as a function of *x*_NaOH_, the fraction of NaOH solution, in ROMB solutions having different proportions of 150 mM NaOH solution to 150 mM Na_2_CO_3_ solution. One part of the ROMB solution is added to nine parts of acidified canine blood. Dashed lines represent parameter values of the acidified canine blood prior to adding the ROMB solution. Solid line in **(A)** is the average of the measured pH values; solid lines in **(B–D)** are linear least squares fits (see text for parameter values and uncertainties).

*(1 part solution: 9 parts CA-K9-2): see Figure 3 for plot of added strong base solutions. Two-component solutions are designated by a non-indented first component and an indented second component in the column under Added solution*.

**Table 2:** **Responses of HCl-CA acidified canine blood to added base solutions**.

**Added base solution**	**Conc. (mM)**	**pH**	**PCO_2_ (mm Hg)**	**[${\text{HCO}}_{3}^{-}$] (mM)**	**[Na^+^] (mM)**	**[K^+^] (mM)**	**[Cl^−^] (mM)**
None	0	6.62	113	10.8	128	3.8	94
NaHCO_3_	150	6.82	141	21.2	135	3.1	92
Na_2_CO_3_	150	7.31	61	28.4	153	3.0	98
NaOH	150	7.21	23	8.3	135	3.2	95
Na_2_CO_3_ + NaOH (1:1)	75 + 75 = 150 tot	7.37	28	15.3	143	3.1	97

**Figure 4:**
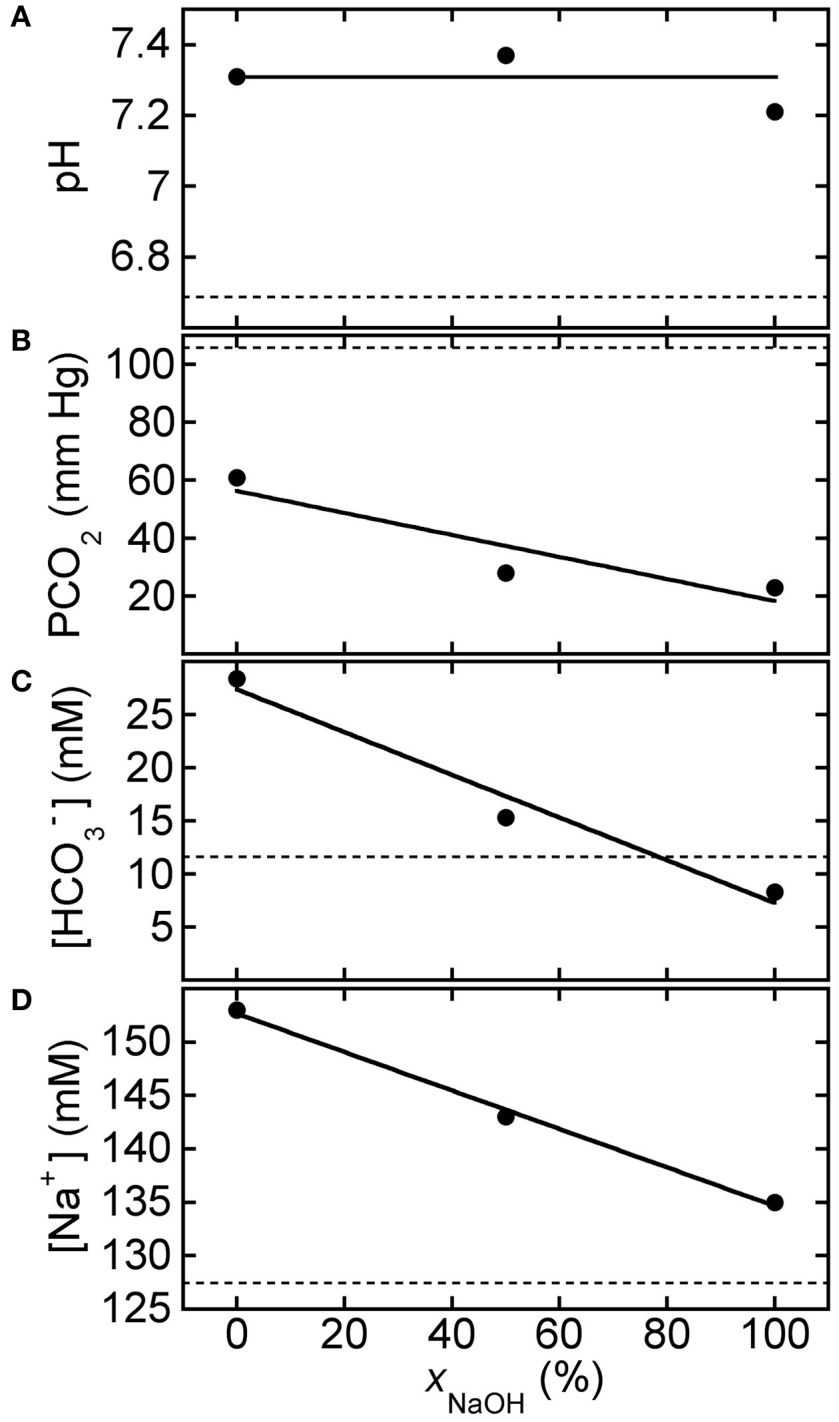
**Response of HCl-acidified canine blood to ROMB solutions composed of Na_2_CO_3_ and NaOH**. Measured blood gas parameters **(A)** pH, **(B)** PCO_2_, **(C)** [${\text{HCO}}_{3}^{-}$], and **(D)** [Na^+^] of HCl-CA-K9-2 acidified canine blood as a function of *x*_NaOH_, the fraction of NaOH solution, in ROMB solutions having different proportions of 150 mM NaOH solution to 150 mM Na_2_CO_3_ solution. One part of the mixed solution is added to nine parts of acidified canine blood. Dashed lines represent parameter values of the acidified canine blood prior to adding the ROMB solution. Solid line in **(A)** is the average of the measured pH values; solid lines in **(B–D)** are linear least squares fits (see text for parameter values and uncertainties).

*(1 part solution: 9 parts HCl-CA-K9-2): see Figure 4 for plot of added strong base solutions*.

**Table 3:** **Responses of HCl-CA acidified canine blood to added base solutions: concentration dependence at fixed 1:1 mixing ratio of NaHCO_3_ and Na_2_CO_3_**.

**Added base solution**	**Conc. (mM)**	**pH**	**PCO_2_ (mm Hg)**	**[${\text{HCO}}_{3}^{-}$] (mM)**	**[Na^+^] (mM)**	**[K^+^] (mM)**	**[Cl^−^] (mM)**
None	0	6.62	113	10.8	128	3.8	94
NaHCO_3_ Na_2_CO_3_	167 167	7.76	38	50.5	171	2.7	106
NaHCO_3_ Na_2_CO_3_	333 333	*8.36*	13	−	−	2.6	118

**Figure 5:**
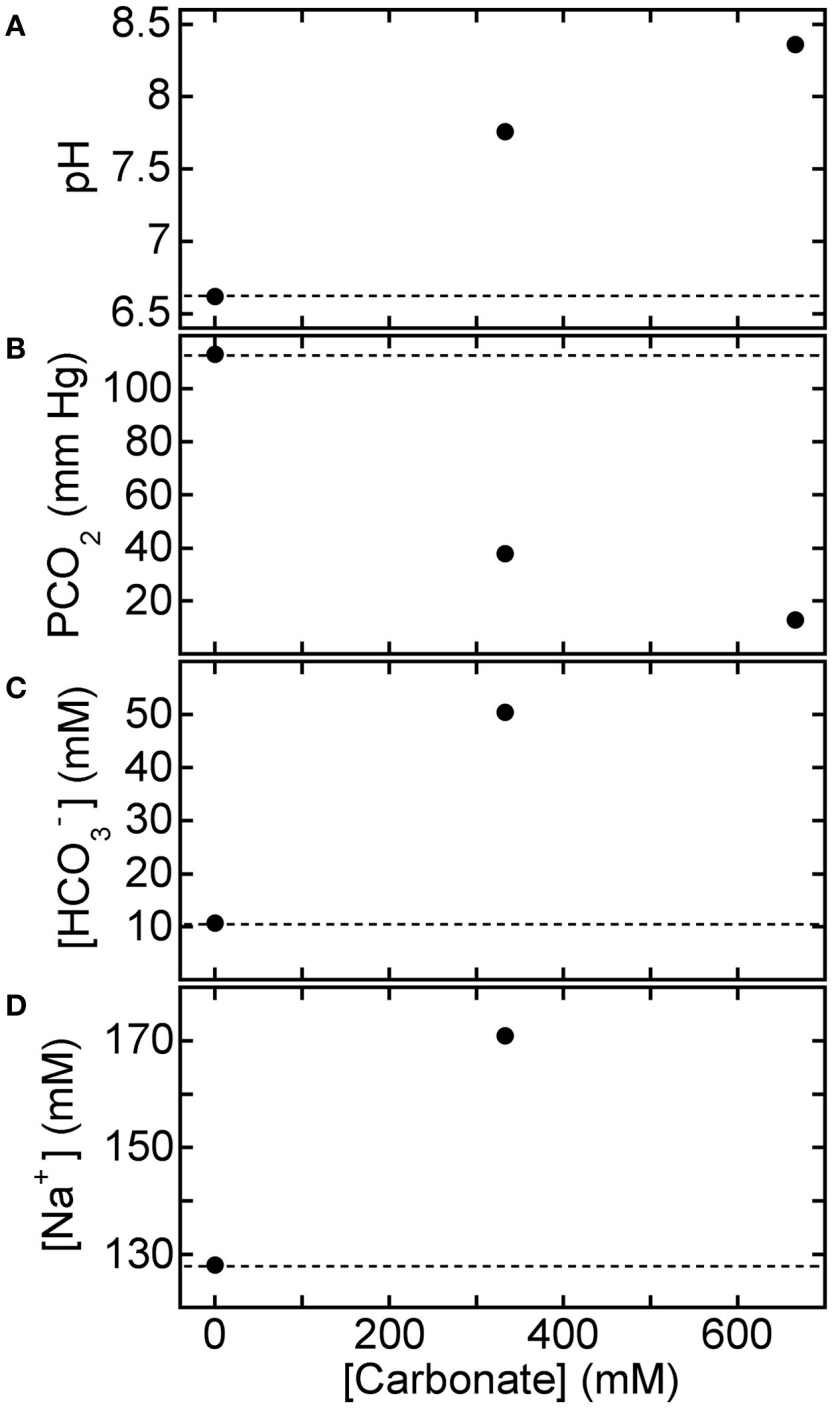
**Response of HCl-acidified canine blood to 1:1 solutions composed of NaHCO_3_ and Na_2_CO_3_**. Measured blood gas parameters **(A)** pH, **(B)** PCO_2_, **(C)** [${\text{HCO}}_{3}^{-}$], and **(D)** [Na^+^] of HCl-CA-K9-2 acidified canine blood vs. concentration of carbonate species, [Carbonate], of equal volumes of aqueous solutions of equimolar NaHCO_3_ and Na_2_CO_3_ (i.e., 1:1 proportions). One part of the mixed base solution is added to nine parts of acidified canine blood. In each panel, the reference parameter value of acidified blood without base treatment corresponds to the point at [Carbonate] = 0 mM and the dashed line. Values for [${\text{HCO}}_{3}^{-}$] and [Na^+^] at the highest concentration lie beyond the detection limits of the BGA.

*(1 part solution: 9 parts HCl-CA-K9-2): see also Figure 5 for corresponding plot*.

*A pH value in italics represents a non-BGA electrode measurement*.

*Dashes indicate values outside of the instrumental range of the BGA*.

**Table 4:** **Responses of CA-acidified canine blood to added base solutions: dependence on mixing ratio of NaHCO_3_ and Na_2_CO_3_ at fixed 150 mM carbonate concentration**.

**Added base solution**	**Conc. (mM)**	**pH**	**PCO_2_ (mm Hg)**	**[${\text{HCO}}_{3}^{-}$] (mM)**	**[Na^+^] (mM)**	**[K^+^] (mM)**	**[Cl^−^] (mM)**
None	0	6.98	75	16.4	148	3.5	98
NaHCO_3_ Na_2_CO_3_	0 150	7.54	44	34.7	162	2.8	103
NaHCO_3_ Na_2_CO_3_	25 125	7.50	47	34.3	162	2.8	102
NaHCO_3_ Na_2_CO_3_	37.5 112.5	7.54	47	36.9	162	2.8	101
NaHCO_3_ Na_2_CO_3_	75 75	7.41	57	33.6	158	2.9	99

*(1 part solution: 9 parts CA-K9-1)*.

**Table 5:** **Responses of HCl-CA acidified canine blood to added base solutions: dependence on mixing ratio of NaHCO_3_ and Na_2_CO_3_ at fixed 150 mM carbonate concentration**.

**Added solution**	**Conc. (mM)**	**pH**	**PCO_2_ (mm Hg)**	**[${\text{HCO}}_{3}^{-}$] (mM)**	**[Na^+^] (mM)**	**[K^+^] (mM)**	**[Cl^−^] (mM)**
None	0	6.66	115	11.9	130	3.0	95
Na_2_CO_3_	150	7.39	47	26.2	152	2.5	99
NaHCO_3_ Na_2_CO_3_	25 125	7.31	60	27.4	152	3.6	99
NaHCO_3_ Na_2_CO_3_	37.5 112.5	7.20	73	26.4	150	4.3	99
NaHCO_3_ Na_2_CO_3_	75 75	7.04	101	25.2	145	5.6	98
NaHCO_3_	150	6.86	135	22.6	136	2.6	93
NaOH NaCl	150 75	7.33	21	10.1	143	2.6	100
Tris soln NaCl	150 75	7.24	28	11.0	131	2.6	100
Tris buffer	150	6.96	42	8.8	122	2.7	98

*(1 part solution: 9 parts HCl-CA-K9-3): see also **Figure 7** for plot of top panel. Lower panel: responses caused by adding other non-carbonate base solutions*.

**Table 6:** **Measured pH of base solutions**.

**Base solution**	**Measured pH**
150 mM NaHCO_3_	8.18
1.0 M NaHCO_3_	8.10
75 mM Na_2_CO_3_	11.18
100 mM Na_2_CO_3_	11.33
150 mM Na_2_CO_3_	11.42
200 mM Na_2_CO_3_	11.35
250 mM Na_2_CO_3_	11.35
360 mM Na_2_CO_3_	11.37
1.0 M Na_2_CO_3_	11.66
100 mM NaOH + 100 mM NaCl	12.91
150 mM NaOH	12.95
75 mM Na_2_CO_3_ + 75 mM NaOH	12.64
150 mM Tris + 75 mM NaCl	10.08
150 mM Tris Buffer	8.11
167 mM NaHCO_3_ + 167 mM Na_2_CO_3_	9.80
333 mM NaHCO_3_ + 333 mM Na_2_CO_3_ (i.e., Carbicarb)	9.63
25 mM NaHCO_3_ + 125 mM Na_2_CO_3_	10.48
37.5 mM NaHCO_3_ + 112.5 mM Na_2_CO_3_	10.23

Figure 3 shows BGA results for pH, PCO_2_, [${\text{HCO}}_{3}^{-}$], and [Na^+^] after three different 150 mM strong-base solutions, containing disodium carbonate and sodium hydroxide in different proportions but fixed molarity (150 mM Na_2_CO_3_ only, 1 part 150 mM NaOH mixed with 1 part 150 mM Na_2_CO_3_, and 150 mM NaOH only), were added to CA-treated canine blood (CA-K9-2). We use the dimensionless mole fraction of NaOH, *x*_NaOH_, in the strong-base mixture as a quantitative identification. All three strong-base solutions provide about the same increase in pH of the CA-treated blood, since both strong bases generate about the same concentration of hydroxide ions and the overall molarity of strong-base species has been fixed at 150 mM, regardless of *x*_NaOH_. We calculate the average pH = 7.50 ± 0.06 of the base-treated blood (solid line in Figure 3A), so the increase in pH caused by the strong base treatment from the reference level (dashed line) is ΔpH = 0.60 ± 0.06. However, PCO_2_ is more substantially reduced by using a base solution that contains more NaOH than Na_2_CO_3_. A linear least squares fit of the results for PCO_2_ in Figure 3B yields a slope of −0.20 ± 0.03 mm Hg/%*x*_NaOH_ and an intercept of 44.0 ± 2.2 mM Hg at *x*_NaOH_ = 0% (correlation coefficient *R* = 0.985). Likewise, a linear fit of [${\text{HCO}}_{3}^{-}$] in Figure 3C yields a slope of −0.22 ± 0.03 mM/%*x*_NaOH_ and an intercept of 36.4 ± 1.6 mM (*R* = 0.993). While NaOH provides essentially the same pH increase as Na_2_CO_3_, the equilibrium sodium ion concentration in the treated blood is lower for NaOH than for Na_2_CO_2_ because NaOH is monovalent in sodium but Na_2_CO_3_ is divalent in sodium. A linear least squares fit of the results for [Na^+^] in Figure 3D yields a slope of −0.17 ± 0.01 mM/%*x*_NaOH_ and an intercept of 164.2 ± 0.4 mM (*R* = 0.999). All reported uncertainties are plus or minus one-standard deviation; high values of *R* near unity indicate that linear fits are appropriate for the observed trends.

In a related experiment for HCl-CA-treated blood, Figure 4 shows BGA results for pH, PCO_2_, [${\text{HCO}}_{3}^{-}$], and [Na^+^] after 150 mM strong-base solutions, containing NaOH only, Na_2_CO_3_ only, and 50% NaOH mixed with 50% Na_2_CO_3_, were added to HCl-CA-K9-2. The trends in the results with *x*_NaOH_ are similar to those in Figure 3, even though the starting pH of HCl-CA-K9-2 is lower and its starting PCO_2_ is higher as a consequence of the additional HCl-acidification of the CA-treated blood. In Figure 4A, the average pH of the strong-base-treated acidified blood is 7.30 ± 0.08, so the increase in pH as a result of treatment with the strong base mixture is ΔpH = 0.59 ± 0.08, comparable to that in Figure 3A. A larger proportion of NaOH causes a greater reduction in PCO_2_, which could be desirable in principle. A linear least squares fit of the results for PCO_2_ in Figure 4B yields a slope of −0.38 ± 0.16 mm Hg/%*x*_NaOH_ and an intercept of 56.0 ± 10 mM Hg at *x*_NaOH_ = 0% (*R* = 0.920). Likewise, a linear fit of [${\text{HCO}}_{3}^{-}$] in Figure 4C yields a slope of −0.20 ± 0.03 mM/%*x*_NaOH_ and an intercept of 27.4 ± 2.3 mM (*R* = 0.985). When pure NaOH is used, [${\text{HCO}}_{3}^{-}$] drops below its initial concentration, and this would typically be undesirable if the initial bicarbonate ion concentration in the acidified blood is already below the normal range. In Figure 4D, [Na^+^] decreases linearly as more NaOH, which contains less sodium, replaces Na_2_CO_3_ in the added solution. A linear least squares fit of the results for [Na^+^] in Figure 4D yields a slope of −0.18 ± 0.01 mM/%*x*_NaOH_ and an intercept of 152.7 ± 0.7 mM (*R* = 0.998). The slopes of all of the decreasing trends in Figures 3B–D, 4B–D are similar, within the uncertainties, so the changes in responses of the BGA parameters to the mixed strong-base solutions are similar regardless of the degree of pre-acidification of the blood. For HCl-CA-treated blood, a value of *x*_NaOH_ = 20% yields desirable BGA values for pH, PCO_2_, [${\text{HCO}}_{3}^{-}$], and [Na^+^] in the treated blood that are all in the normal range. Thus, by controlling the relative proportions of carbonate and non-carbonate bases in a mixed base solution, it is possible to raise pH in a similar manner to a desirable level, yet control the carbonate parameters in the base-treated acidified blood independently so as to achieve desirable levels of PCO_2_ and [${\text{HCO}}_{3}^{-}$] also.

For reference, Figure 5 shows BGA results after mixing equimolar 1:1 base-treatment solutions, which contain equal proportions of the weak base NaHCO_3_ to the strong base Na_2_CO_3_ but different total carbonate species concentrations, with HCl-CA-K9-2 (9 parts acidified blood: 1 part base solution). Reference values of the BGA parameters of the acidified canine blood in the absence of base treatment correspond to points at [Carbonate] = 0 mM. The effects of Carbicarb treatment are given by the points at a total carbonate concentration of 667 mM (i.e., at the largest [Carbonate] shown). While we find that the Carbicarb composition does raise pH and lower PCO_2_ considerably, it also raises [${\text{HCO}}_{3}^{-}$] and [Na^+^] dramatically, and these effects could potentially be undesirable in treatment applications, beyond concerns related to Carbicarb's potential for creating adverse hypertonic effects because of its high molarity.

**Figure 6:**
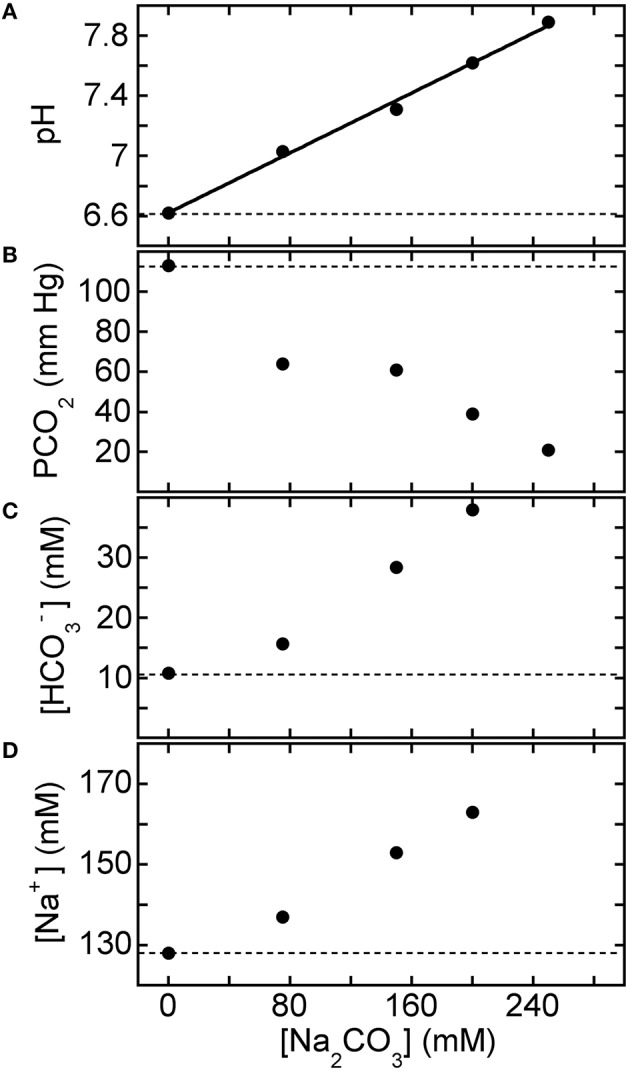
**Response of HCl-acidified canine blood to Na_2_CO_3_ solutions: concentration dependence**. Measured blood gas parameters **(A)** pH, **(B)** PCO_2_, **(C)** [${\text{HCO}}_{3}^{-}$], and **(D)** [Na^+^] of HCl-CA-K9-2 acidified canine blood as a function of [Na_2_CO_3_] of added base solution. One part of disodium carbonate solution is added to nine parts of acidified canine blood. In each panel, the reference parameter value of acidified blood without base treatment corresponds to the point at [Na_2_CO_3_] = 0 mM and the dashed line. Solid line in **(A)** is a linear least squares fit (see text).

BGA results are plotted in Figure 6 after adding disodium carbonate base solutions at different concentrations to HCl-CA-K9-2. Reference values of the BGA parameters of the acidified canine blood in the absence of base treatment correspond to points at [Na_2_CO_3_] = 0 mM. The pH-response of the treated blood to [Na_2_CO_3_] in the treatment solution is linear (*R* = 0.997); the slope from the least squares fit in Figure 6A is 5.0 ± 0.2 pH unit/M and the intercept is at pH = 6.62 ± 0.03. The addition of Na_2_CO_3_ also causes a significant reduction in PCO_2_ while [${\text{HCO}}_{3}^{-}$] rises. However, as a simple consequence of stoichiometry, using Na_2_CO_3_ causes [Na^+^] to rise with added carbonate concentration twice as fast as an equivalent molarity solution of NaHCO_3_. Consequently, [Na^+^] rises out of the normal range at the highest values shown. If only Na_2_CO_3_ is used to treat HCl-CA-acidified canine blood, then the most optimal concentration to bring all four BGA parameters shown into the normal range is about 150 mM Na_2_CO_3_.

**Figure 7:**
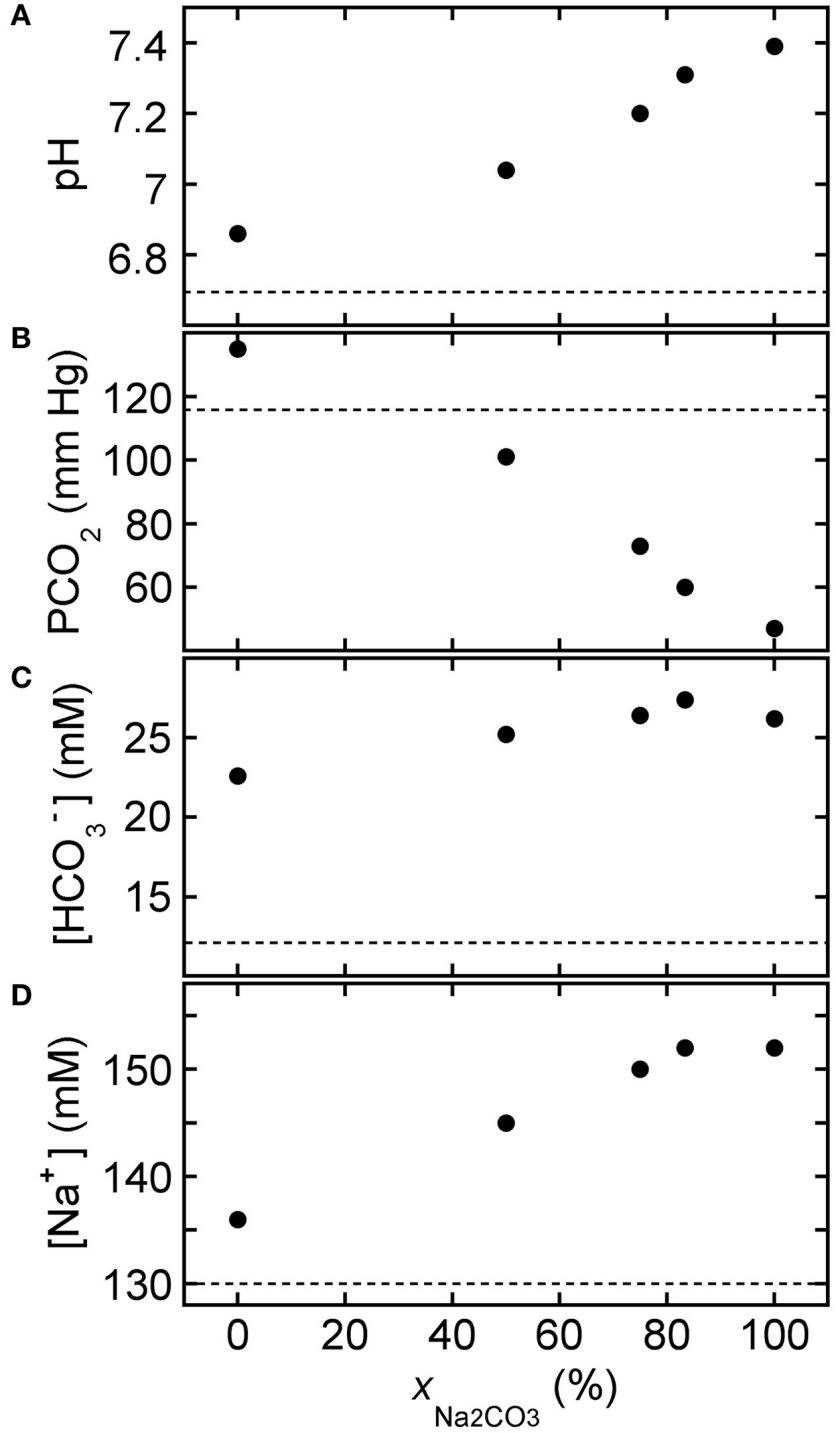
**Response of HCl-acidified canine blood to ROMB solutions composed of NaHCO_3_ and Na_2_CO_3_**. Measured blood gas parameters **(A)** pH, **(B)** PCO_2_, **(C)** [${\text{HCO}}_{3}^{-}$], and **(D)** [Na^+^] of HCl-CA-K9-3 acidified canine blood as a function of *x*_Na_2_*CO*_3__, the fraction of Na_2_CO_3_ solution in a ROMB solution made by mixing a 150 mM NaHCO_3_ solution with a 150 mM Na_2_CO_3_ solution in different proportions. One part of ROMB solution is added to nine parts of acidified canine blood. Values at *x*_Na_2_*CO*_3__ = 75% correspond to the 3:1 ROMB solution. Dashed lines represent measured values for the acidified canine blood prior to the addition of the ROMB solution.

By contrast, Figure 7 shows BGA results after ROMB solutions containing differing proportions of NaHCO_3_ to Na_2_CO_3_, yet a fixed total added carbonate species concentration of 150 mM, were added to HCl-CA-K9-3. As the percentage of Na_2_CO_3_ increases, the pH of the treated blood rises significantly while PCO_2_ is lowered. Moreover, [${\text{HCO}}_{3}^{-}$] can be kept in a desirable range. Although there is an increase in [Na^+^], it is not as extreme as with hypertonic base solutions such as 1.0 M sodium bicarbonate or Carbicarb. In particular, a 3:1 ROMB solution, made by mixing three parts 150 mM Na_2_CO_3_ to one part 150 mM NaHCO_3_, offers a desirable increase in pH while reducing PCO_2_ and maintaining [${\text{HCO}}_{3}^{-}$] and [Na^+^] near the normal range.

**Figure 8:**
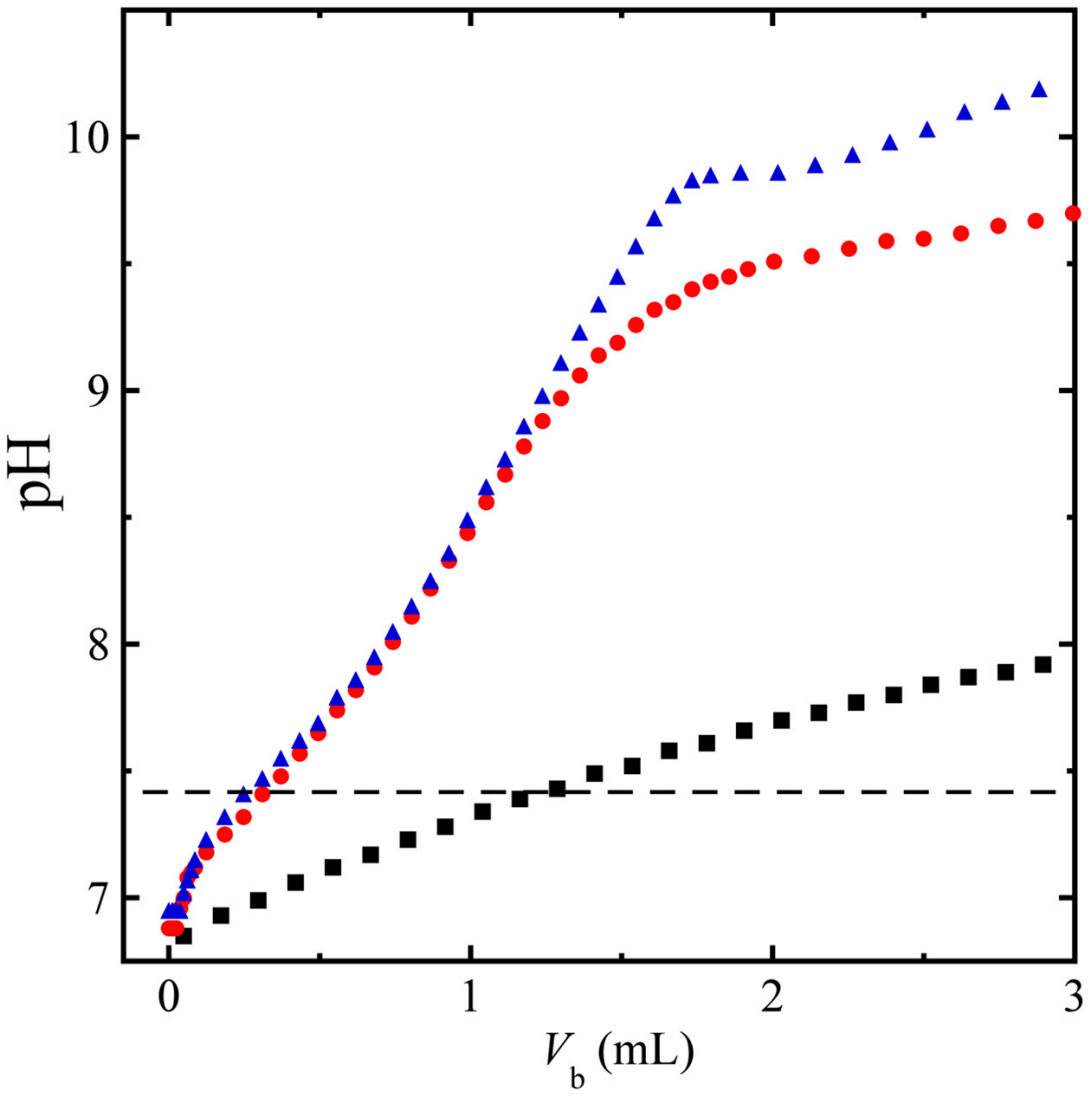
**Titration of HCl-acidified canine blood by base solutions**. Measured pH during titration of an initial volume of 4 mL of HCl-CA-K9-3 acidified canine blood using an added volume *V*_b_ of a base solution, 150 mM NaHCO_3_ (squares), 100 mM Na_2_CO_3_ (circles), and 100 mM NaOH + 100 mM NaCl (triangles); Dashed line, pH = 7.4 reference level, showing that nearly 5 times the volume of 150 mM NaHCO_3_ would be needed compared to 100 mM Na_2_CO_3_ solution to raise the pH of the blood to the reference level. The titration has been performed at 23°C.

To complement BGA measurements, we have also performed separate titrations of acidified canine blood HCl-CA-K9-3 using three different base solutions (150 mM NaHCO_3_, 100 mM Na_2_CO_3_, and 100 mM NaOH: 100 mM NaCl) while measuring pH using the pH meter, as shown in Figure 8. A volume *V*_b_ of base solution is mixed with an initial volume of 4.0 mL of HCl-CA-acidified canine blood. The ROMB solutions containing strong bases Na_2_CO_3_ and NaOH/NaCl yield nearly identical titration curves for small added base volumes *V*_b_ ≤ 1 mL. In this case, relatively small volumes of these solutions, *V*_b_ ≈ 0.25 mL, raise pH into a desirable range to about 7.4; by contrast, a much larger *V*_b_ ≈ 1.2 mL of the base solution of the weak NaHCO_3_ is needed. Thus, ROMB solutions containing a large proportion of a weak base would be significantly less effective in raising pH of acidified blood. For the two strong base solutions shown, interesting non-linear responses are seen at higher *V*_b_, but such large relative administration volumes would not be practical in clinical applications.

### Optical microscopy of blood treated using mixed-base solutions

**Figure 9:**
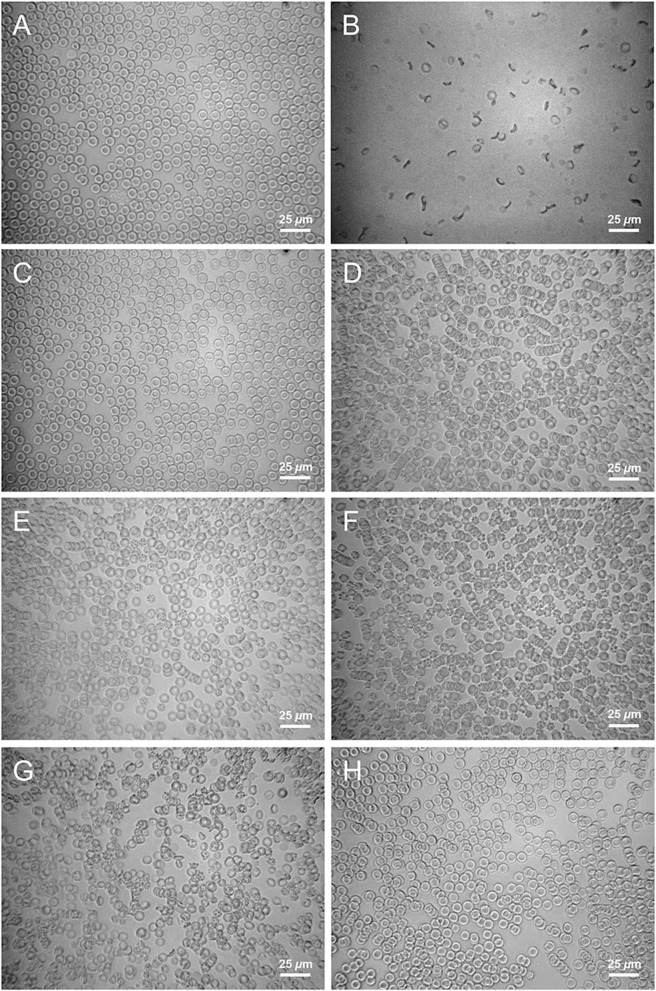
**Micrographs of acidified canine blood after mixing with solutions**. Optical brightfield transmission micrographs of 9 parts CA-treated canine blood: 1 part solution. **(A)** CA-treated blood without any added solution (control), **(B)** 1.0 M NaHCO_3_, **(C)** 150 mM Na_2_CO_3_, **(D)** 150 mM NaOH, **(E)** 75 mM Na_2_CO_3_ + 75 mM NaOH, **(F)** 150 mM HCl, **(G)** 333 mM NaHCO_3_ + 333 mM Na_2_CO_3_, **(H)** 150 mM NaOH + 75 mM NaCl.

We have also investigated the consequences of adding mixed-base solutions to CA-treated blood using brightfield optical transmission microscopy, which can reveal spiculation, lysis, and rouleaux formation (Baskurt et al., 1997; Bäumler et al., 1999) of red blood cells (RBCs). For reference, Figure 9A shows a microscope image of CA-treated canine blood as received from the supplier. The RBCs in fresh CA-treated blood have a normal appearance (biconcave, diameter ≈ 7 μm). Consequently, CA-treated blood serves as a useful reference point for optical microscopy observations: any change in the structures of RBC's seen in CA-treated blood that has been mixed with a base solution are clearly a consequence of that particular base solution. Because a significant percentage of RBCs in HCl-CA-treated blood exhibit spiculation and rouleaux formation (see Figure 9F), which are complex and possibly irreversible structural changes, even before mixing with a base solution, HCl-CA-treated blood is less useful as a starting point for determining structural changes caused by base treatment through optical microscopy experiments. Thus, our optical microscopy experiments focus primarily on mixtures of CA-treated blood with base solutions.

Figure 9B shows an example of the appearance of 9 parts CA-treated blood mixed with 1 part of a 1.0 M NaHCO_3_ solution, which is approved for use in medical practice to treat acute forms of acidosis. The image shows that considerable damage can be caused to RBCs, presumably by osmotic effects, as a result of adding 1 part of hypertonic 1.0 M treatment solution to 9 parts of blood and rapidly mixing it. Based on this finding, where a 1.0 M NaHCO_3_ solution first contacts the blood of a patient (e.g., where the base solution exits the IV needle and enters the blood), it is possible that at least some RBCs and other structures in the blood would be damaged, even if a large population would remain undamaged because of flow mixing and dilution with the patient's much larger blood volume. Thus, very rapid IV administration of a hypertonic 1.0 M sodium bicarbonate solution could cause some undesirable osmotic damage to blood cells and create additional stress on an acidotic patient.

By contrast, Figure 9C shows an example of the appearance of canine blood that has been treated with a solution of 150 mM Na_2_CO_3_. The RBCs are clearly biconvex and are not spiculated; they appear very similar to those in Figure 9A and are essentially undamaged. This demonstrates that a reduced osmolarity solution of a strong base, in this case disodium carbonate, can be mixed into blood without causing visible damage to RBCs. Thus, the strong base solution of 150 mM Na_2_CO_3_ does effectively raise pH and lower PCO_2_ in blood; plus, it does not visibly damage RBCs. Figure 9D shows an example of the appearance of canine blood that has been treated with a reduced osmolarity solution of 150 mM NaOH. Relatively little damage to RBCs is observed; a few (i.e., less than 10%) spiculated RBCs are seen. However, rouleaux formation is evident. For certain species of mammals, blood that has been removed and not flowing is known to exhibit spontaneous rouleaux formation (e.g., in equine blood) (Baskurt et al., 1997; Bäumler et al., 1999). Thus, blood can still be functional and viable even if rouleaux are seen. So, the observation of rouleaux formation of RBCs, while not ideal, is of minor importance compared to changes in the shape and integrity of RBCs.

Reducing the amount of NaOH and increasing the amount of Na_2_CO_3_, yet keeping the total composition near isotonic concentration, can reduce the rouleaux formation. Figure 9E shows an example of the appearance of canine blood that has been treated with a ROMB solution of 75 mM NaOH + 75 mM Na_2_CO_3_, yielding a total concentration of strong base species of 150 mM (i.e., having a similar power to raise pH as solutions in Figures 9C,D). Rouleaux formation is largely absent; only a few very small aggregates are seen. Although a very small population (i.e., less than 10%) of spiculated RBCs are observed, the vast majority of RBCs are undamaged, similar to Figure 9C.

CA-treated canine blood that has been mixed with 150 mM HCl (9 parts blood: 1 part HCl solution) is shown in Figure 9F. A significant population (approximately 30%) of the RBCs exhibit spiculation, and rouleaux are also observed. Because these features are irreversible and could affect the interpretation of microscope images of HCl-acidified blood that is subsequently treated with base solutions, we do not show any images of base-treated HCl-CA-acidified canine blood.

Figure 9G shows an optical micrograph of CA-acidified canine blood that has been treated with Carbicarb. A large fraction, nearing about 50%, of RBCs are damaged: noticeably spiculated, deformed, or broken. Some smaller rouleaux are also observed of the remaining biconcave fraction of RBCs. This micrograph, combined with Figure 9B, provides evidence that rapid injection of highly concentrated (i.e., very hypertonic) base solutions is unlikely to be optimal when treating acute forms of acidosis.

Acidified canine blood CA-K9-3, treated with a ROMB solution of saline-supplemented sodium hydroxide (150 mM NaOH + 75 mM NaCl added in 1 part to 9 parts blood), is shown in Figure 9H. Nearly all RBCs are normal and biconcave and very few are spiculated. A few smaller rouleaux are present, but the addition of a small saline concentration appears to reduce the amount of spiculation and also rouleaux formation, compared to 150 mM NaOH only (see Figure 9C).

## Discussion

Our *in silico* study provides a fundamental basis for understanding ROMB solutions, which could potentially be customized and used to treat different types of acute acidoses. A key finding, in accord with thermodynamic principles, is that reducing the overall osmolarity of a base solution while also using a higher proportion of a strong base, can still be effective in raising blood pH while lowering PCO_2_ and maintaining or increasing blood [${\text{HCO}}_{3}^{-}$] concentration. This higher proportion of strong base produces hydroxide ions, ultimately consuming carbonic acid and therefore reducing PCO_2_, by Le Chatelier's principle and Henry's Law (Oxtoby et al., 2012). In addition, our predictions show that in some circumstances strong monosodium bases, such as NaOH, could potentially offer an advantage over strong disodium bases, such as Na_2_CO_3_, because monosodium bases reduce added [Na^+^]. Based on our calculations, we hypothesize that the chemical composition of a base solution could be customized and optimized for individual acidotic patients according to the types and severities of acidoses as well as the potential need for a reduced sodium load. We expect that using non-ideal activities, rather than ideal concentrations of species in these equations, such as are given by the Debye-Hückel or Davies equations (Tissue, 2013), would improve the accuracy of the calculated predictions but would not affect the overall trends.

Our *in vitro* measurements indicate that ROMB solutions that contain majority proportions of a strong base in their compositions could be beneficial compared to conventional hypertonic weak base solutions and other existing alternatives. Optimizing the composition of a ROMB solution can be performed with the goal of raising blood pH as well as that of interstitial and intracellular compartments, lowering blood and tissue CO_2_, and minimizing potential osmotic damage to circulating blood elements. For instance, ROMB solutions containing a higher proportion of a strong base, (e.g., 3:1 Na_2_CO_3_:NaHCO_3_), by contrast to hypertonic 1:1 Carbicarb, appear to hold promise as efficacious treatment solutions for certain types of acidosis. Through optical microscopy, we have also demonstrated that hypertonic 1.0 M sodium bicarbonate solution and hypertonic Carbicarb, if administered rapidly, could potentially cause osmotic damage to blood cells. By contrast, ROMB solutions, particularly those that have an osmolarity closer to isotonic, perturb cellular structures significantly less than such hypertonic solutions when mixed rapidly. Thus, ROMB solutions such as 3:1 Na_2_CO_3_:NaHCO_3_ could potentially be used to advantage, compared to existing hypertonic solutions, in situations where raising pH rapidly without causing significant damage to beneficial components in the blood is indicated. If a lower sodium load is desired, then a certain fraction of Na_2_CO_3_ could be replaced by NaOH in a ROMB mixture with NaHCO_3_, but this may need to be moderated in consideration of the propensity of NaOH to reduce [${\text{HCO}}_{3}^{-}$] compared to Na_2_CO_3_. Beyond this, in certain clinical situations, treatment to achieve a lower blood pH range between about 7.2 and 7.3 can be desirable; in such cases, using a ROMB solution that has a ratio lower than 3:1 strong base:weak base (e.g., a 2:1 solution) could potentially enable different desired lower pH targets to be reached with reasonable injection volumes while further reducing risk of possible caustic damage. Thus, through compositional customization, ROMB solutions appear to offer some limited but interesting potential for controlling pH as well as other electrolyte and dissolved gas parameters into desired target ranges simultaneously.

Future studies, performed both *in vitro* and *in vivo*, could examine the impact of rate-controlled titrations of blood using various base solutions and lead to additional insight into how the composition and rate of administration of base solutions can be tailored most beneficially for the treatment of certain acid-base disorders. Regarding *in vitro* experiments, investigating the effects of the history of microcirculation and pH change on the structures of RBCs and rheology of blood would be interesting to pursue. Regarding *in vivo* experiments, optimizing ROMB solutions for the treatment of animals with metabolic or respiratory acidosis could lead to significantly improved outcomes. In such studies, it would be desirable to measure intracellular pH_i_ and extracellular pH_e_ in certain tissues, not just arterial blood pH. There might be conditions for which administration of bicarbonate solutions could raise the pH of cells rather than reduce it (Nielsen et al., 2002). Also, the normal pH-regulatory proteins, including the Na^+^-H^+^ exchanger, NHE1, will tend to improve cellular acid-base balance even in the absence of administered base restoring cellular pH to baseline. However, activation of this transporter can cause deleterious increments in cellular sodium and calcium and typically needs to be minimized (Wu and Kraut, 2014). Thus, there are likely to be some situations in which administration of simple bicarbonate solutions might be sufficient without requiring the properties of a ROMB solution that contains a high proportion of a strong base. Measuring the deformability of RBCs as a function of pH (Kuzman et al., 2000), by adjusting the composition and concentration of added base solutions at different flow conditions, including in microfluidic channels (Korin et al., 2007), would likely provide a quantitative basis for understanding how to control and alleviate local caustic and osmotic damage to RBCs and other structures.

Indeed, it is highly unlikely that a single base solution would be optimal for treating all of the many different types of acute acidoses that are encountered clinically in a wide variety of patients who have varied genetics and other co-existing medical conditions. Our work on ROMB solutions instead suggests that an optimal composition, concentration, and method of administration of a base solution for a particular patient could potentially be custom-tailored according to the severity and nature of the patient's acid-base parameters, his/her physiological parameters, other co-existing medical conditions, and possibly also, if known, genetics of important acid-base transporters and other proteins. We emphasize that our *in silico* and *in vitro* studies have not accounted for active processes, such as changes in respiration, bicarbonate production, and acid production, which in and of themselves can significantly affect the acid-base milieu of tissues. Therefore, extending our theoretical modeling to account for the influence of ROMB solutions on complex time-dependent transport reactions in active open systems that mimic organisms with circulation and respiration, as well as including a broader range of ionic species, is a promising future direction. Such future predictive time-dependent models could incorporate additional ionic complexity and organ-related sources and sinks inherent in Stewart's classic “strong ion difference” approach (Stewart, 1983), including divalent cations and poly-electrolytic proteins, as well as complex non-diffusive and diffusive transport.

In summary, from a clinical perspective, because measures to eliminate the underlying cause of the acidosis are not always successful, administration of base to improve the acid-base parameters is often recommended. However, the most common base administered, sodium bicarbonate, is often associated with acidification of the intracellular compartment, even if it improves systemic acid-base balance. Because this acidification can be a consequence of the CO_2_ generated through the buffering process, measures to improve acid-base balance without generating CO_2_ would potentially be of great value. The only base that is clinically available which can accomplish this task is THAM. However, THAM has been associated with certain adverse effects, and THAM requires intact renal function to be effective. Our present studies have therefore examined the impact that various strong bases and ROMB solutions can have on pH, [${\text{HCO}}_{3}^{-}$], and PCO_2_ when added in realistic proportions to acidified blood. The results indicate that certain base combinations can potentially improve acid-base balance and actually reduce CO_2_ generation. These particular bases would likely be most effective in treating patients with acute lactic acidosis due to reduced tissue perfusion, a common cause (Kraut and Madias, 2016). They could also be potentially useful in treating patients with acute respiratory acidosis that have an impaired ability to expel CO_2_ actively. Testing the viability of ROMB solutions in the treatment of acidotic animals, which could have different controlled types and severities, and then eventually humans seems to be indicated based on promising features of these solutions, as surmised from these initial studies.

## Author contributions

TM and JK: Designed and conducted *in vitro* experiments; TM: Derived equations, wrote computer programs, and performed *in silico* calculations; TM and JK: Analyzed experimental results and wrote the manuscript.

## Funding

This work was supported by UCLA.

### Conflict of interest statement

The authors are inventors of provisional US patent application 14/420,861, US patent application US 2015/0196708 A1, and PCT patent application PCT/US2013/055629, all assigned to UCLA. As UCLA employees, the authors are required to disclose potentially patentable research to UCLA's Office of Intellectual Property, which makes independent decisions regarding filings. US 2015/0196708 A1 contains an example Mathematica code (see Figure 14 of this US patent application) for solving the equations presented herein. The authors have received no financial compensation to date as a consequence of this disclosure or these filings.
